# Determinants of Green Innovation to Achieve Sustainable Business Performance: Evidence From SMEs

**DOI:** 10.3389/fpsyg.2021.767968

**Published:** 2021-11-18

**Authors:** Yasser Baeshen, Yasir Ali Soomro, Muhammad Yaseen Bhutto

**Affiliations:** ^1^Department of Marketing, Faculty of Economics and Administration, King Abdulaziz University, Jeddah, Saudi Arabia; ^2^Department of Marketing, Business School, Shandong Jianzhu University, Jinan, China

**Keywords:** green absorptive capacity, green innovation, sustainable human capital, organization support, economic performance, environmental performance, social performance, firm size

## Abstract

Environmental degradation and global warming are major challenges to humankind in the twenty-first century. Thus, businesses are now adopting and incorporating more sustainable manufacturing methods to produce environmental products and services. It is inevitable for organizations to adopt green practices and achieve sustainable performance. This extant research addresses how to obtain sustainable development (SD) through green innovation (GRIN). The main purpose of this study is to develop a comprehensive model by integrating natural resource-based view (NRBV) and triple bottom line (TBL) framework. Three antecedents namely green absorptive capacity (GAC), sustainable human capital (SHC), and organization support (OS) were selected, and their influence was checked on GRIN of the SMEs from manufacturing sector. This study included all three factors of TBL: environmental, economic, and social sustainability in terms of GRINs possible consequences. Data were randomly collected from 304 firms in the kingdom of Saudi Arabia through questionnaire. Convergent and discriminant validity analyses were conducted to assure validity and reliability, and structural equation modeling (SEM) was utilized to assess the relationships between variables using smartPLS 3.0 software. Further, firms were categorized into two groups based on company size—small and medium—to explore group differences. Hence, firm size was included as a moderator in the proposed model and multi-group analysis (MGA) was performed. The results indicate that GAC, SHC, and OS have positive influence on GRIN within SMEs. Further, results reveal GRIN had strong significant impact on all three variables of sustainable performance. The study concludes with MGA results that provided evidence of significant group differences, with a stronger relationship between GAC and GRIN in medium-sized firms compared to small-sized firms. Similarly, the relationship between GRIN and environmental performance was stronger in medium-sized firms than small-sized firms. This study is unique and provides practical and theoretical implications. This paper offers an integrative model for sustainability which may be of interest to scholars, marketers, and policymakers.

## Introduction

Environmental degradation is causing major challenges to humankind, their economic success, and nature (Leonidou et al., 2017). Due to the growing concern of environmental issues, governments and businesses are concentrating on more sustainable manufacturing methods and integrating sustainable processes into core business operations (Das and Rangarajan, 2020; Muangmee et al., 2021; Qu et al., 2021; Huang et al., 2019). Environmental challenges have also highlighted the importance of small- and medium-sized enterprises (SMEs). SMEs also play a significant role in creating jobs, manufacturing value added products, and driving innovations to local economies (OECD, 2017). Hence, SMEs are generally perceived as the backbone of the economy. On the other hand, SMEs often account for more than 60–70% of industrial pollution because these companies are numerous and less focused on environmental protection (Hillary, 2004). According to researchers’ recommendations and policymakers, one of the most effective techniques for SMEs to reduce pollution while maintaining competitiveness is “GRIN” (Jun et al., 2019).

In comparison with large enterprises, SMEs are extremely resistant to technological change and more adaptive to market changes, while their organizational structure enables them to make quicker decisions (Pilar et al., 2018). SMEs have lately begun to embrace green innovation (GRIN) initiatives in response to stakeholder pressures (Jun et al., 2019). However, the adoption of GRIN in SMEs is still unknown (Aboelmaged and Hashem, 2019). Innovation studies, particularly those focusing on SMEs, have attempted to explain and examine how to foster an atmosphere conducive to innovation and identify the key factors of organizational innovation. Still, the innovation process, the capabilities and resources inside a firm that foster GRIN, as well as the relationship between the two, remain complex. While several organizational capabilities and factors exist, numerous studies have missed important capabilities in their research. A holistic and integrated approach is thus required to transform SMEs in emerging markets (Aboelmaged and Hashem, 2019).

Kingdom of Saudi Arabia (KSA) is the world’s largest oil producer (Balat, 2006). Despite fast industrial and economic progress, KSA has encountered major environmental difficulties, including air pollution, energy waste, and water pollution (Raggad, 2018). According to the Ministry of Labor and Social Development, Saudi Arabian SMEs generate around 22% of the Kingdom’s GDP. Approximately 34% of Saudi employees work in (SMEs; Jadwa Investment, 2019). The kingdom has adopted the Vision 2030 strategic plan to transform the economy and stimulate innovation in the country; as a result, SMEs are aided in their efforts to develop eco-friendly manufacturing processes. To fill the gap in the literature on emerging markets’ promising challenges of sustainable economic development and environmental conservation, with a particular emphasis on Saudi Arabia. This study makes three significant contributions: first, the internal and external organizational factors analyze GRIN adoption. A research model is built to demonstrate how green absorptive capacity (GAC), sustainable human capital (SHC), and organizational support (OS) affect GRIN in SMEs in Saudi Arabia.

Secondly, how GRIN affects the firm to obtain sustainable performance. The majority of past research has focused on performance as a single dimension (Xie et al., 2019) or has taken two dimensions of organization performance (i.e., Economic and Satisfaction; Yáñez-Araque et al., 2017; Hernández-Perlines et al., 2019). The authors of this study have used the Triple bottom line (TBL) model to view performance as a multidimensional term (i.e., social, economic, and environmental performance; Shahzad et al., 2020). Thus, the following three SP dimensions were investigated in this study: environmental, economic, and social sustainability. Thirdly, this study used firm size as a moderating variable to investigate the group difference between medium-sized and small-sized SMEs.

## Theoretical Background and Hypotheses Development

The intersection of the natural resource-based view (NRBV) and TBL framework provides the theoretical foundation for this study. It is argued that the effectiveness of GRIN adoption is influenced by organizational capabilities derived from the firm’s NRBV (Aboelmaged and Hashem, 2019). According to the NRBV (Hart, 1995), the competitiveness of a company can be maintained through the use of strategic VIRO (value, difficult to copy, unique, and organized) resources (Lin and Wu, 2014). According to the NRBV, a firm’s environmental abilities, stewardship of products and services, and overall sustainability all add to its competitiveness (Hart, 1995). A firm’s response to global environmental changes can foster a sustainable capability. It reflects the firm’s environmental capabilities and resources (Teece, 2009) and can provide proactive solutions to sustainability issues. NRBV helps meet stakeholders’ environmental concerns while also providing other advantages, such as energy conservation, environmental recycling, material reduction, pollution prevention, etc. (Chen et al., 2014; Wang, 2019). Following Minbashrazgah and Shabani (2019) research, they considered three interrelated internal practices: SHC, sustainable orientation, plus sustainable collaboration can positively drive GRIN.

Additionally, researchers also reveal that absorptive capacity (AC) which is a firm’s capability does influence GRIN (Arfi et al., 2018). AC reflects the firm’s ability to recognize and use external knowledge. AC is increasingly seen as critical to innovation and competitive performance (Danquah, 2018). March and Simon (1958) found that rather than developing new information and experiences, most innovative firms identify and absorb them from other organizations. After that, Cohen and Levinthal (1990) refined the concept further, arguing that firms’ AC reflects their ability to recognize, integrate, and implement valuable external knowledge.

The environmentalist and naturalists endorsed the organizations for incorporating advanced knowledge and green ideas into their manufacturing processes to benefit increased business sustainability (Qu et al., 2021). Similarly, sustainable development (SD) has been addressed in current environmental management literature. Though, this is a hot topic, academics and experts are not yet in agreement on the concept and definitions (Hahn et al., 2015). The World Commission on Environment and Development (WCED, 1987) defined SD as “development that meets the needs of the present without compromising the ability of future generations to meet their needs.” Economic, environmental, and social challenges are all included in this definition of WCED. These three pillars of sustainable business performance (SBP), together referred to as the “triple bottom line”, affect present and future generations (Elkington, 1998). TBL is a CSR framework with three dimensions: economic, social, and environmental. These three dimensions must achieve sustainable goals. Each of the three dimensions of sustainable performance (environmental, economic, and social) is rather prominent in this framework; consequently, it may be considered an integrative theory of sustainability (Asadi et al., 2020).

Studies found that SBP plays a vital role in meeting SD goals using various strategies together such as corporate social responsibility (CSR), GRIN, and AC (Abbas, 2020; Shahzad et al., 2020). However, the influence of organizational factors such as GAC, SHC, and OS combine has not been investigated to achieve sustainable performance in SMEs through GRIN. Moreover, prior empirical studies on absorptive capacity, internal capabilities, and GRIN are precisely uncommon, especially in developing nations and SMEs (Albort-Morant et al., 2018; Aboelmaged and Hashem, 2019). Furthermore, few studies can be found to integrate NRBV and TBL frameworks in SMEs in developing contexts. To fill these gaps is vital for advising SMEs on innovation policies. Thus, this study aims to bridge the gap by adopting NRBV and TBL framework in the context of SMEs.

### Green Innovation

GRIN is a term that refers to technological advancements that are used to manage the environment, prevent pollution, reduce waste, and conserve energy (Chen, 2008; Zhang et al., 2019). GRINs help businesses function better by reducing waste and costs for a sustainable environment (Kleindorfer et al., 2005). Additionally, GRIN increases market positions, builds brands, spurs innovation, and attracts potential customers (Chandy and Tellis, 2000). GRIN is intrinsically linked to corporate environmental management and environmental goal attainment. As a result, it is often considered that GRIN results in increased performance (Zailani et al., 2015). Few recent studies have revealed that GRIN is a key factor that directly affects sustainable business performance (Abbas and Sağsan, 2019; Shahzad et al., 2020). Numerous prior studies have demonstrated the effect of GRIN on performance (Gluch et al., 2009; Arfi et al., 2018). Numerous organizational variables are examined concerning GRIN adoption, including human resource quality, top manager leadership skills, OS, and organizational culture (García-Machado and Martínez-Ávila, 2019; Jun et al., 2019). This study focuses on GAC, OS, and SHC, as these elements consistently have a greater impact on GRIN adoption (Zailani et al., 2015; Aboelmaged and Hashem, 2019).

### Green Absorptive Capacity and Green Innovation

Organizations require dynamic capabilities, such as absorptive capacity, to keep up with the rapidly changing environment (Cohen and Levinthal, 1990; Martinez-Sanchez and Lahoz-Leo, 2018). The literature on innovation identifies a firm’s absorptive capacity as the main factor driving innovation (Ali and Park, 2016; Ali et al., 2020). Earlier research has established that adopting innovative practices in manufacturing or service contexts requires a firm’s capacity to acquire, transmit, and apply internal and external knowledge (Tseng and Hung, 2011). For example, Tseng and Hung (2011) found that knowledge transfer and absorptive capacity positively affected organizational innovation and performance in Taiwan’s knowledge-intensive business sectors. Albort-Morant et al. (2018) suggested that a firm’s AC enhances its ability to generate innovative methods that have a positive environmental impact, like green products, services, or processes. They confirmed that firms are aware of external knowledge detailing how their businesses environmental issues, such as pollution, waste, and other results, negatively affect the world, and integrate it with their existing knowledge to assist in adopting GRIN methods. These methods may include developing new environment-friendly products and procedures for waste reduction, recycling, pollution control, and more.

On the other hand, GAC is a very new and untested concept (Chen et al., 2014). Chen et al. (2015) defined “green absorptive capacity” as a high capacity for identifying, assimilation, and utilization of external knowledge. The GAC of a firm can help developing a sustainable competitive advantage through the use of external and internal green knowledge (Qu et al., 2021). As a result, we may say that an organization wanting to increase its GRIN performance should put an emphasis on the firm’s GAC. Green absorptive capacity enables a business to identify, acquire, integrate, and utilize environmental knowledge in order to foster GRIN (Chen et al., 2014). GAC enables enterprises to effectively manage environmental knowledge, enhancing firms’ GRIN capabilities (Chen et al., 2015; Roberts, 2015). Further, Chen et al. (2015) used structural equation modeling (SEM) to study the relationship between absorptive capacity and green services in the context of the electronic industry in Taiwan. They concluded that absorptive ability had a positive effect on service-oriented GRIN. Organizations require the green absorptive capability to generate creative and innovative ideas from environmental knowledge to develop a sustainable competitive advantage through GRIN (Chen et al., 2015). Additionally, GAC aids in disseminating environmental knowledge across various departments (Roberts, 2015), thereby strengthening firms’ interaction mechanisms, research and development, and management processes (Vicente-Oliva et al., 2015), thus further enhancing GRIN. Recently, Qu et al. (2021) revealed and endorsed that GAC is a key factor contributing to GRIN. Thus, SMEs must integrate external and internal knowledge to embrace new environmentally friendly methods to achieve GRIN. Based on the discussion above, our study proposes the following hypothesis:

Hypothesis 1: GAC positively influences GRIN within SMEs.

### Sustainable Human Capital and Green Innovation

The availability of skilled human capital and managers’ dedication to environmentally friendly activities are also considered as major factors motivating businesses to create environmentally friendly practices (Wu et al., 2012). According to Huang and Kung (2011), sustainable human resources can display important abilities such as an environmentally friendly direction, attitude, skills, knowledge, competence, and knowledge through employee activities. GRIN can be fostered by key intangible and tangible assets, as well as the knowledge of environmental friendly management and staff (Aboelmaged and Hashem, 2019). The HR function can encourage SHC by means of green practices in recruitment, training, and by awarding green initiatives (Renwick et al., 2016). As per Consoli et al. (2016), many green jobs require more training, education, work expertise, cognitive abilities, and interpersonal skills than non-green jobs. In addition, many workers in the United States have expressed an interest in working for green employers (Gully et al., 2013). SHC can help organizations reduce uncertainty, accept risk, and overcome employee opposition to innovation and green practices (Chen and Chang, 2013). The availability of skilled human resources in-house and managers’ adherence to green practices are regarded as the primary factors pushing businesses to pursue GRIN (Lee, 2008; Wu et al., 2012). Additionally, Lin and Ho (2011) stated that rewarding employees for eco-friendly conduct could encourage workers to adopt green practices. Support from top management is critical for achieving sustainable goals, particularly support for employees’ green behavior, making organizational resources easily accessible to employees, improving the quality of recruitment, learning capabilities of the organization, and accumulating environmental knowledge (Lin and Ho, 2011). Previous studies have revealed that SHC have a significant positive impact on the adoption of GRIN (Cowden et al., 2015; Singh et al., 2020). Still, there is a need to probe more on SHC within the context of sustainability (Yong et al., 2019). As a result, the authors anticipate that SHC will positively affect GRIN.

Hypothesis 2: SHC positively influences GRIN within SMEs.

### Organizational Support and Green Innovation

The term “organizational support” refers to the degree to which an organization facilitates its employees in using a certain technology or system (Naujokaitiene et al., 2015). Fostering innovation and assuring the availability of innovative funding and technical resources significantly affect innovation adoption (Clohessy and Acton, 2019). OS is critical for the development of environmental management because it ensures that the resources necessary to implement green practices are readily available and that workforce is motivated to do so. Above all, top management has a significant role in providing OS. Most green practices require coordination and collaboration between various departments of an organization during implementation. To ensure their success, green projects are typically supported and encouraged by higher management. The key task of top management is to ensure resource availability and distribute them proficiently so that the firm can adopt green practices to gain a competitive environmental advantage (González-Benito and González-Benito, 2006). Hence, we anticipate that OS will have a positive impact on SMEs GRIN and offer the following hypothesis:

Hypothesis 3: OS positively influences GRIN within SMEs.

### Green Innovation and Sustainable Business Performance

The TBL model highlighted the importance of the economy, society, and environment as the dimensions of firm performance (Elkington, 1998; Asadi et al., 2020). This study has included all three dimensions from the perspective of SMEs, as these are critical for sustainable innovation and business performance (Asadi et al., 2020). In line with this, scholars have pointed out the importance of financial performance, social welfare, and environmental quality in the general public’s well-being (Mahrinasari and Bangsawan, 2020; Shahzad et al., 2020). Ali et al. (2016) discover a favorable relationship between a firm’s innovativeness and overall sustainable performance. Further, Rajapathirana and Yui (2018) found a positive correlation between the innovativeness of SMEs and their overall performance (Ali et al., 2020). However, scholars argue that organizations are more focused on the economic element than social and environmental (Asadi et al., 2020). For the successful operations of the business, all components have a crucial role in the success of business performance (Fernando et al., 2019).

Environmental performance (ENP) can be described as the environmental impact of a firm’s green activities (Henriques and Sadorsky, 1996; Chen, 2008). GRIN practices can reduce environmental threats (e.g., air emissions, regularity of landfill disposal) to enhance a firm’s environmental performance (Kammerer, 2009), and its reputation in the industry (Dangelico, 2013). Environmental performance is assessed by firms that minimize waste generation and carbon dioxide emissions, and the use of harmful chemicals (Asadi et al., 2020; Muangmee et al., 2021). Previous research has shown that the introduction of green technologies will increase the chances of improving environmental performance (Singh et al., 2016; Xue et al., 2019). As a result, the following hypothesis was proposed:

Hypothesis 4a: GRIN positively affects the ENP of SMEs.

GRIN does benefit businesses that outperform their competitors. Companies are generally engaged in GRIN activities to support a diverse range of transactions that suit the demands and needs of potential buyers. This can result in increased sales volume, which enhances a company’s financial situation (Chen, 2008; Caracuel and Ortiz-de-Mandojana, 2013; Zhang et al., 2020). Many studies show that more innovative organizations tend to do better financially. For example, Marques and Ferreira (2009) found that innovativeness gives firms a competitive advantage, improving their financial performance. Economic success is also a result of GRIN. Numerous businesses are beginning to create next-generation clean technologies to help their economic development in the future. Shell has been investing in solar, wind, and other renewable energy sources to believe that they can quickly substitute non-renewable energy (Hart and Milstein, 2003). According to Xie et al. (2019), GRIN mitigates negative environmental impacts and enhances economic performance (ECP) through reduced waste and costs. Furthermore, cost savings in production are essential for GRIN and a sustainable environment (Hojnik and Ruzzier, 2016), and Li et al. (2017) revealed the positive influence of GRIN on ECP. As a result, the following hypothesis was presented in this study.

Hypothesis 4b: GRIN positively affects the ECP of SMEs.

Apart from solving environmental problems, GRINs are critical for employee retention, improving communication, and increasing the brand’s acceptability. Development of human capital through training may assist in persuading employees and shifting their behavior and attitude toward more environmentally friendly practices (Huang et al., 2016). It turns out that being environmentally aware and having good environmental practices both positively affect corporate profits and employee well-being. Additionally, it provides several other benefits, including increased social responsibility, recruitment, and retention of qualified individuals (Mehta and Chugan, 2015; Muangmee et al., 2021). Indeed, as Wagner (2013) indicates, the performance of organizations that invest in social accountability, pay proper attention to satisfied customers through innovation and hire appropriately qualified employee’s increases. Previous research indicates that businesses’ green performance increases their social performance (SOCP; Asadi et al., 2020; Shahzad et al., 2020). Based on prior research, the following hypothesis is developed:

Hypothesis 4c: GRIN positively affects the SOCP of SMEs.

### Firm Size as Moderator

There is much literature on the factors of innovation and, in particular, on the impact of firm size on innovation (Acs and Audretsch, 1991). The firm’s size has been consistently identified as a significant organizational element influencing environmentally friendly behavior (Etzion, 2007) and performance (Hernández-Perlines and Xu, 2018). Generally, big corporations implement environmentally friendly practices more quickly than small firms, given their considerable resources and essential infrastructure (Lin and Ho, 2010). Previous literature reveals that small firms are significantly less inventive than big companies (Roper and Hewitt-Dundas, 1998; Raymond and St-Pierre, 2010). For example, Du et al. (2007) discovered that when internal resource factors were considered in firms with 10 or more employees, size had a favorable effect on the likelihood of innovation, but at a declining rate. Sdiri and Ayadi (2011) observed a similar impact when examining the effects of the same determinants on the decision to innovate using simultaneous and sequential models applied to a dataset of 108 Tunisian service firms. This is because, while large companies may benefit from economies of scale in terms of technology and learning, such benefits may be overshadowed by organizational differences in size (Zenger, 1994). Likewise, according to Vossen (1998), “the relative strength of large firms is largely determined by their financial resources and technology, while the relative strength of small firms is primarily determined by their entrepreneurial dynamism, flexibility, efficiency, proximity to the market, and motivation.” Within the framework of SMEs, this study used SMEs as moderators to see if there are significant differences and implications for the adoption of GRIN.

### The Conceptual Model

**Figure 1:**
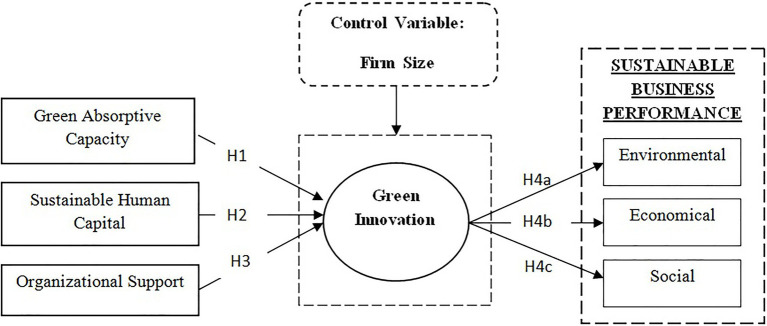
Research model.

The research model of the study in Figure 1 shows the impact of GAC, SHC, and OS on GRIN. Further, it depicts the effect of GRIN on SBP and its all dimensions (environmental, economic and social) following the TBL. Moreover, the moderating role of firm size is also examined.

## Methodology

### Data Collection Procedure and Participants

A primary research design with a quantitative approach was performed to empirically interpret the proposed conceptual hypothesis developed and presented in the framework. The data for this study were gathered in two phases: first, semi-structured interviews with five managers were conducted. These managers worked in SME manufacturing companies in the Jeddah industrial area in Saudi Arabia to identify the most important drivers of GRIN and sustainable performance. A research questionnaire was developed by adopting construct items used in earlier literature on GRIN and SBP. In this study, the data were gathered using a random sampling technique. Because each unit has the equal chance, random sampling is considered the most appropriate sampling approach (Secker et al., 1995). The items of constructs were adopted and modified to suit the current research. Green absorptive capacity had five items developed by Chen et al. (2014). For the organization factor, two sub-constructs were included: SHC (Chang and Chen, 2012; Aboelmaged and Hashem, 2019) and OS (Jun et al., 2019). GRIN had four items modified from previous studies (Kusi-Sarpong et al., 2015; Aboelmaged and Hashem, 2019). Lastly, sustainable business performance had three constructs: environmental performance having three items (Bansal, 2005; Wang, 2019), economic performance having three items (Bansal, 2005), and social performance had four items (Bansal, 2005). All items were slightly modified to suit our study on SMEs. The finalized questionnaire was spread to SMEs belonging to six industry sectors: Construction, Energy, Logistics, Manufacturing, IT, and Services. A total of 304 valid responses were received, and all the responses were included for data analysis, among 304 respondents from the industry, comprised of entrepreneurs (33.1%), senior management (41.4%), and middle management (25.3%). Female respondents dominated (60.5%) in the sample compared to male respondents (39.5%). In Saudi Arabia, SMEs are defined as businesses that employ 249 or fewer employees and have less than SR200m ($53.3m) in annual revenue. As per Saudi Nitaqat data and the General authority for statistics (GaStat), authors categorize firms into two sizes: small firms having 6–49 employees and medium firms with 50–249 employees. Out of 304 responses, 189 were from medium-sized firms, and the remaining 115 were from small firms. The demographic analysis of the data collected from managers of Firms can be seen in Table 1.

**Table 1:** Demographic analysis.

Demographics		Frequency	Percent
Gender	Female	184	60.5
	Male	120	39.5
Industry/Sector	Construction	64	21.1
	Energy	40	13.2
	IT	47	15.5
	Logistics	41	13.5
	Manufacturing	56	18.4
	Services	56	18.4
Job level	Entrepreneur	101	33.2
	Middle management	77	25.3
	Senior management	126	41.4
Firm size	Medium (50–249 employees)	189	62.2
	Small (6–49 employees)	115	37.8

## Results

The gathered data were analyzed using Partial Least Squares Structural Equation Modeling (PLS-SEM; Hair et al., 2013, 2014); the PLS-SEM analysis gave the measurement model analysis which includes validity measurements given below in Table 2.

**Table 2:** Factor loadings, composite reliability, and average variance extracted.

Constructs	Codes	Loadings^a^	Cα	rho_A	CR	AVE^b^
Green absorptive capacity			0.959	0.959	0.959	0.825
	GAC1	0.897				
	GAC2	0.937				
	GAC3	0.893				
	GAC4	0.902				
	GAC5	0.910				
Sustainable human capital			0.934	0.935	0.934	0.781
	SHC1	0.896				
	SHC2	0.908				
	SHC3	0.897				
	SHC4	0.833				
Organization support			0.917	0.918	0.917	0.786
	OS1	0.866				
	OS2	0.876				
	OS3	0.917				
Green innovation			0.931	0.932	0.932	0.773
	GIA1	0.874				
	GIA2	0.902				
	GIA3	0.866				
	GIA4	0.874				
Economic performance			0.907	0.907	0.907	0.766
	ECP1	0.878				
	ECP2	0.880				
	ECP3	0.867				
Social performance			0.723	0.897	0.801	0.559
	SOCP1	0.876				
	SOCP2	0.869				
	SOCP3	0.843				
	SOCP4	(0.072 deleted)				
Environmental performance			0.890	0.898	0.890	0.732
	ENP1	0.772				
	ENP2	0.941				
	ENP3	0.845				

*Cα, Cronbach’s Alpha; CR, composite reliability; rho_A, Dijkstra-Henseler’s rho; AVE, average variance extracted*.

a *All loadings are significant at p<0.001*.

b *Percentage of variance of item explained by the construct*.

### Measurement Model

Table 2 shows that the measurement model had good convergent validity. Evaluating convergent validity through examining (AVE) of each latent construct. The average variance extracted value greater than 0.5 indicates that the validity of the variable and construct is high. According to Sarstedt (2017), items loading must lie between 0.05 and 0.07; it was observed that out of 25 items, one item that is related to social performance (our organization recognized and acted on the need to fund local community initiatives) was deleted because it is outer loadings was below 0.07. Hence, in the whole model, only 24 items were retained as loadings greater than 0.07 (see Table 2), indicating that the measurement model was reliable and meaningful.

Discriminating validity was observed by comparing the indicators with other reflective indicators in the cross-loading. The authors followed the Fornell and Larcker rule of thumb (Fornell and Larcker, 1981). Regarding discriminant validity, to achieve the discriminate validity, the square root of the AVE should be higher than the correlation among latent variables (Henseler et al., 2018). In Table 3, the correlation among the construct was compared to the square root of the AVE (values in boldface). The outcome generated with the help of SmartPLS3 and Excel shows that the square root of AVE was all greater than the correlations among the constructs, revealing adequate discriminant validity.

**Table 3:** Co-relation matrix.

	ECP	ENP	GAC	GRIN	OS	SOCP	SHC
Economic performance	**0.875**						
Environmental performance	0.846	**0.855**					
Green absorptive capacity	0.820	0.814	**0.908**				
Green innovation	0.957	0.933	0.855	**0.879**			
Organization support	0.874	0.890	0.883	0.858	**0.887**		
Social performance	0.992	0.965	0.816	0.955	0.822	**0.748**	
Sustainable human capital	0.831	0.801	0.953	0.888	0.860	0.654	**0.884**

*Entries shown in boldface represent the square root of the AVE (average variance extracted). GAC, green absorptive capacity; SHC, sustainable human capital; OS, organizational support; GRIN, green innovation; ENP, environmental performance; ECP, economic performance; and SOCP, social performance*.

### Structural Model

**Figure 2:**
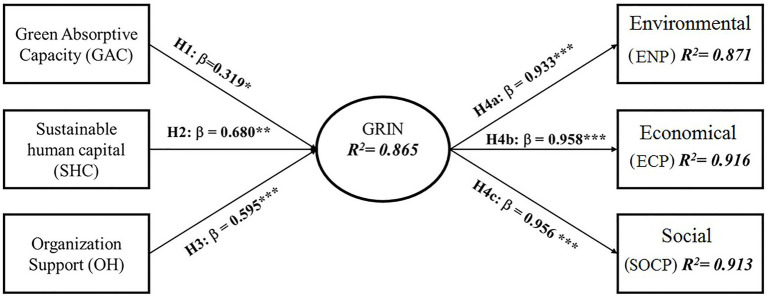
Structural model results with beta values, ^***^ denotes *p*<0.001, ^**^p<0.01, ^*^p<0.05.

The bootstrapping technique was used in this study to determine the relevance and importance of each hypothesis. The findings are presented in Table 4 of statistically significant hypotheses acquired using the bootstrapping technique (304 responses, 5,000 samples with no sign change option). To confirm the relevance of hypotheses, t-statistic was utilized with two-tailed test (Hair et al., 2017). The results of the hypotheses testing show that all of the proposed hypotheses are supported (Table 4). According to the statistical results of PLS-SEM, GACE has a positive and significant impact on GRIN (H1; *β*=0.319; *p*<0.05), SHC also has positive and significant impact on GRIN (H2; *β*=0.680; *p*<0.01) and similarly OS (H3; *β*=0.595; *p*<0.001), thus supporting H1, H2, and H3. Finally, GRIN has a positive and significant impact on ENP (H4a; *β*=0.933; *p*<0.001), ECP (H4b; *β*=0.958; *p*<0.001) and SOCP (H4c; *β*=0.956; *p*<0.001); thus H4a, H4b and H4c are accepted. Detailed results are also displayed in Figure 2. Additionally, the standardized root means square residual (SRMR) value is 0.044, less than the threshold limit of 0.08, indicating an excellent model fit. The SRMR index was used to verify the overall goodness-of-fit score for structural model validation in this study (Hair et al., 2017).

**Table 4:** Hypothetical relationships.

Hypothesis	Structural path	Standardized path coefficient	*t*-value	Significant difference (*p* <0.05)?	Findings
H1	GAC → GRIN	0.319	1.372	**0.042** ^*^	Supported
H2	SHC → GRIN	0.680	3.379	**0.001** **	Supported
H3	OS → GRIN	0.595	5.706	**0.000** ***	Supported
H4a	GRIN → ENP	0.933	47.105	**0.000** ***	Supported
H4b	GRIN → ECP	0.958	35.785	**0.000** ***	Supported
H4c	GRIN → SOCP	0.956	34.367	**0.000** ***	Supported

*GAC, green absorptive capacity; SHC, sustainable human capital; OS, organizational support; GRIN, green innovation; ENP, environmental performance; and ECP, economic performance*.

* *p < 0.05*,

** *p < 0.01*,

*** *p < 0.001*.

#### Multi-Group Analysis (Firm Size)

An MGA was used to examine the impact of the variables between the different groups. There are four techniques to analyze these groups, according to Sarstedt et al. (2013): parametric, permutation, confidence-based, and Henseler’s multi-group approach. Henseler (2012) provided an even more developed adaptation for the latter, the PLS-MGA technique (Multi-Group Analysis), which indicates significant differences between groups when they are less than 0.05 or greater than 0.95. The authors employed a percentile bootstrapping method to examine the differences between the two groups of firms in this study. As previously stated, the authors received 304 replies in this survey, with 189 responses coming from medium-sized businesses and the remaining 115 from small businesses. As a result, the authors checked the moderating role of firm size in the model. When the value of *p* was greater than 95 percent or less than 5 percent, the results demonstrated a significant intergroup difference with a 5 percent margin of error. A percentile of less than 5% implies that the bootstrap result for group A (Medium Size firm) is better than that of group B (Small Size Firm). A percentile exceeding 95% shows that the yield of group B is higher than that of group A (Table 5).

**Table 5:** Multi group analysis (firm size).

Relationships	Path (M)	Path (S)	Diff.	PLS MGA (*p*)
H1 GAC → GRIN	0.087	−0.162	0.249	0.045
H2 SHC → GRIN	0.120	0.064	0.056	0.661
H3 OS → GRIN	0.136	0.246	−0.110	0.636
H4(a) GRIN → ENP	−0.171	0.084	−0.255	0.159
H4(b) GRIN → ECP	0.247	0.296	−0.048	0.785
H4(c) GRIN → SOCP	0.940	0.892	0.047	0.013

*GRIN, green innovation; ENP, environmental performance; SOCP, social performance; ECP, economical performance. Bold font: PLS-MGA value of p below 5% and above 95% indicates significant values. Diff., path coefficient differences*.

The PLS-MGA value of *p* results shows that there are significant differences between medium and small SMEs. Our study found a significant difference in H1 (*p*=0.249<0.05), which means that the relationship between GAC and GRIN is stronger in medium-sized firms than in small-size firms. In addition, our study also found a significant difference in H4 (a) (*p*=0.047<0.05), which shows that the relationship between GRIN and environmental performance is stronger in medium-size SMEs in the comparison of small-size SMEs.

## Discussion and Conclusion

This study explores the antecedents and consequences of GRIN in order to gain a better knowledge of the important success factors. Using the conceptual framework presented in Figure 1, this research hypothesizes to examine how GAC, SHC, and OS work together to improve GRIN and to explain the role of GRIN in achieving SBP in manufacturing industries that are trying to remain competitive in the face of changing innovative processes, government legal environmental pressures, and stakeholders demand. The cross-sectional survey results collected from 304 SMEs in Saudi Arabia validate and support all of the hypothesized relationships reported in Table 4.

Our study found that GAC has a significant relationship with GRINs; the results are similar to previous studies that mentioned that GAC enables firms to effectively manage environmental knowledge to improve the ability of firms to adopt GRINs (Chen et al., 2015; Qu et al., 2021). The study results suggest that Saudi manufacturing SMEs manage their core competencies and knowledge resources effectively and efficiently, thereby increasing GRIN and organizational performance. Sustainable human capital has a significant positive impact on GRIN consistent with prior studies (Weng and Lin, 2011; Zailani et al., 2014). The availability of skilled human resources in-house and managers’ adherence to green practices are the primary factors pushing businesses to pursue GRIN (Lee, 2008; Wu et al., 2012). The study results suggest that managers’ commitment to greening SMEs has been demonstrated in the context of Saudi SMEs, which means that this can be achieved when top management of SMEs are fully motivated to pursue GRIN goals. Sustainable human resource has a significant relationship with GRIN; the findings support previous research that determined that SHC positively influences the adoption of GRIN (Cowden et al., 2015; Ortega-Lapiedra et al., 2019; Singh et al., 2020). The results of this research show that the green behavior of employees has a positive effect on GRIN in manufacturing SMEs. In the case of Saudi Arabia, the skills, innovation, abilities, capacity, and responsibility of workers with respect to environmental security promote GRIN in Saudi manufacturing SMEs.

This study found that the adoption of GRINs significantly affects Saudi SMEs’ environmental, economic, and social performance. The results show that the adoption of GRIN can lead to a win-win situation so that SMEs can simultaneously increase their environmental, economic, and social performance. The relationship between GRIN and environmental performance was found significant; the findings of this study support past studies (Weng et al., 2015; García-Machado and Martínez-Ávila, 2019; Singh et al., 2020). The findings of this study suggest that GRIN adoption and better productivity enhance the environmental performance of Saudi manufacturing SMEs. In addition, this study recognizes that GRIN is a critical organizational resource used by SMEs to improve their environmental performance and gain goodwill among industry and stakeholders. The relationship between GRIN and economic performance was found to be significant; this finding supports past studies (Caracuel and Ortiz-de-Mandojana, 2013; Zailani et al., 2015; de Azevedo Rezende et al., 2019). The findings show that Saudi SMEs have realized that GRIN is a critical factor in financial performance. The results suggest that adopting GRINs will reduce pollution, waste, energy, and materials, and process improvements will improve the economic performance of Saudi manufacturing SMEs. The results also support the positive impact on social performance by GRIN, which also supports the stance of previous research conducted by Zailani et al. (2015), such that socially responsible firms are more active and committed to meeting environment-friendly consumer demands to reduce environmental damage (Albort-Morant et al., 2018). The results suggest that the adoption of GRIN will improve the goodwill of Saudi manufacturing SMEs among stakeholders, including employees, customers, and the general public, which will ultimately improve their social performance.

The moderating effect of firm size revealed the significant difference in the relationship between GAC and GRIN. Our study found that compared with small SMEs, medium-sized SMEs have a more substantial impact of GAC on GRIN; medium size SMEs have a better ability to accumulate and utilize knowledge regarding green practices due to their sufficient resources and strong infrastructure. A similar study revealed that large companies adopt GRIN more quickly than small ones (Andries and Stephan, 2019). Further, regarding the environmental performance, this study found a significant difference between medium and small size SMEs; according to the findings of this study, Saudi medium-sized SMEs believe that adopting green technology helps them accomplish environmental performance, which helps advance the firm’s environmental image and CSR.

## Theoretical Implications

This study makes several theoretically contributions. This research offers an unprecedented empirical approach to sustainable business performance in the SMEs industry of KSA by integrating previously separate strands of Innovation theory, Dynamic Capabilities theory, and organizational factors into the Triple-bottom line framework. The results of the study provide the following contributions. First, this study proposed a conceptual model based on NRBV that provides various new correlations to address the lack of prior literature in GRIN. This study proposed model by analyzing several essential subcomponents, such as GAC, SHC, OS on GRIN adoption, and sustainable performance. Such a combination of constructs in a single model is missing in the existing GRIN studies, particularly in manufacturing SMEs. Secondly, Absorptive capacity is a necessary capability for responding to environmental changes in the modern-day. This study establishes that GAC has a significant impact on GRIN and the SBP, both directly and indirectly. Since Cohen and Levinthal (1990) introduced the concept of absorptive capacity, many studies on absorptive capacity have developed within the topic of innovation theory (Ali and Park, 2016; Ali et al., 2020). Although previous research has established the positive effects of absorptive capacity on innovation performance, the role of GAC on GRIN needs more investigation (Chen et al., 2014; Qu et al., 2021). Additionally, just a few academics have applied absorptive capacity to the field of environmental management and GRIN research. Hence, to overcome the gaps in the current literature, this research examined GAC and GRIN in the manufacturing industry within the framework of SMEs. Furthermore, this study also explains how GAC affects GRIN and tries to find a new way to promote GRIN in organizations alongside SHC and OS.

Thirdly, our research found that GRIN is a significant determinant of SBP and all of its dimensions that contribute to protecting the environment and SD; however, GRIN has a strong significant effect on the economic and social dimensions of SBP. As a result, this study adds to the NRBV in a developing economy context by demonstrating that GRIN continues to be an important factor in determining sustainable performance. Furthermore, this empirical study is the first to examine the moderating effect of firm size on GAC, SHC, OS, GRIN, and SBP. According to the results of this study, firm size could also be regarded as a moderator to investigate GRIN and sustainable business performance.

## Practical Implications

This study has numerous practical implications for the managers and policymakers that sustained the competitive advantage of SMEs. First, from the perspective of GAC, this study found that it has a significant impact on the GRIN of the manufacturing SMEs in Saudi Arabia. Firms should increase their efforts to cultivate GAC. They need to develop their absorptive capacity to a strategic and tactical level, develop an internal knowledge management system, and conduct formal and informal training and knowledge sharing activities within departments. Organizations must foster an environment conducive to learning and collaboration throughout the organization in order to encourage and enhance employees’ knowledge acquisition, assimilation, and application capabilities, as well as their creative ability to develop environmentally friendly products, services, and processes, enhance their attentiveness to and awareness of external knowledge and acquire new information and technical resources from external sources. Additionally, competent government authorities should actively promote and guide organizations to connect with and collaborate with academics and scientific research centers. Simultaneously, they should publicize the newest industrial policies and trends, foster collaboration and innovation, and develop knowledge management systems to help local SMEs and entrepreneurial firms improve their overall GRIN performance. Further, senior management, employees, suppliers and customers are well aware of environmental and sustainability issues. The research also helps practitioners and is useful to managers to understand environmental, social and economic performance. In addition, the use of this model in manufacturing in developing countries is expected to improve the ability of organizations to pursue cleaner production and use green human capital as a strategy for achieving sustainable results.

## Limitations and Future Directions

This study has limitations, despite providing unique and valuable insights for scholars and practitioners. The sample was obtained from a one country, therefore limiting the findings’ generalizability to other countries. Future research may include longitudinal surveys from other nations at various phases of development (e.g., developed vs. developing) and from other cultures (e.g., Western vs. Eastern). Additionally, organizational culture may be influenced by national culture, which varies substantially from country to country (Yang et al., 2017). Researchers can compare mean responses and the strength of relationships using such multi-country analysis, contributing to a more holistic understanding of GRIN and its consequences. Additionally, this study covered only manufacturing SMEs, implying that additional research on services and non-manufacturing SMEs is necessary to understand this hot issue better.

## Data Availability Statement

The original contributions presented in the study are included in the article/supplementary material; further inquiries can be directed to the corresponding author.

## Author Contributions

YB, YS, and MB contributed to the conceptualization, formal analysis, investigation, methodology, writing of the original draft, and writing review and editing. All authors contributed to the article and approved the submitted version.

## Funding

This project was funded by the Deanship of Scientific Research (DSR) at King Abdulaziz University, Jeddah, under grant no. (G: 646-120-1441).

## Conflict of Interest

The authors declare that the research was conducted in the absence of any commercial or financial relationships that could be construed as a potential conflict of interest.

## Publisher’s Note

All claims expressed in this article are solely those of the authors and do not necessarily represent those of their affiliated organizations, or those of the publisher, the editors and the reviewers. Any product that may be evaluated in this article, or claim that may be made by its manufacturer, is not guaranteed or endorsed by the publisher.
